# Genesee Valley Veterinary Medical Association

**Published:** 1900-03

**Authors:** E. Knight

**Affiliations:** Secretary


### GENESEE VALLEY VETERINARY MEDICAL ASSOCIATION.

THE annual meeting of this Association was held at the Livingston Hotel, Rochester, N. Y., January 25th. Members present: Drs. W. G. Dodds, A. G. Tegg, L. R. Webber, T. S. Rich, O. B. French, J. H. Taylor, E. Knight, A. T. Earl, W. J. Payn, N. N. Lefler, J. C. McKenzie, L. J. Palmer, A. McConnell, P. J. Johnson, G. C. Kesler, D. P. Webster, William Hunter, and Warren E. Stocking.

Interesting papers were presented by Drs. A. Y. Earl, A. McConnell, A. G. Tegg, L. R. Webber, and N. N. Lefler. During the afternoon session a clinic was held at Webber's livery stable.

Resolutions were passed on the deaths of Drs. E. T. Burns and I. A. Drinkwater. Resolutions were also passed in favor of the enactment of a State law preventing the importation of cattle into New York State without inspection, as follows: *Whereas*, Tuberculosis has and is increasing to an alarming extent among cattle in Western New York, thus causing extensive loss of cattle and endangering the public health; and as most of these cases are in cattle imported from adjoining States or traceable to infection from such cases; *Resolved*, That we, the members of the Genesee Valley Veterinary Medical Association, urgently call for the enactment of such laws as will give us protection and prevent this State from being made the dumping ground for diseased animals that cannot be sold in adjoining States, where they have laws preventing the importation of such diseased animals; and that we urge each member of this Association to call upon his representative in Assembly and Senate to urge the passage of such laws.

Elected to membership: Dr. J. E. Smith, Webster, N. Y.

The following officers were elected: President, Dr. W. G. Dodds; Vice-President, T. S. Rich; Secretary, E. Knight; Treasurer, L. R. Webber. Censors: Drs. A. G. Tegg, O. B. French, G. C. Kesler, L. J. Palmer, W. E. Stocking, and A. McConnell.

E. Knight,
Secretary.

